# Long-term outcome of enzyme-replacement therapy in advanced Fabry disease: evidence for disease progression towards serious complications

**DOI:** 10.1111/joim.12077

**Published:** 2013-05-06

**Authors:** F Weidemann, M Niemann, S Störk, F Breunig, M Beer, C Sommer, S Herrmann, G Ertl, C Wanner

**Affiliations:** Department of Medicine, Divisions of Cardiology and Nephrology, University of WürzburgWürzburg, Germany; Comprehensive Heart Failure Center, University of WürzburgWürzburg, Germany; Institute of Radiology, University of WürzburgWürzburg, Germany; Department of Neurology, University of WürzburgWürzburg, Germany

**Keywords:** dialysis, Fabry disease, prognosis, stroke, sudden cardiac death, α-galactosidase A

## Abstract

**Objective:**

The long-term effects of enzyme-replacement therapy (ERT) in Fabry disease are unknown. Thus, the aim of this study was to determine whether ERT in patients with advanced Fabry disease affects progression towards ‘hard’ clinical end-points in comparison with the natural course of the disease.

**Methods:**

A total of 40 patients with genetically proven Fabry disease (mean age 40 ± 9 years; *n* = 9 women) were treated prospectively with ERT for 6 years. In addition, 40 subjects from the Fabry Registry, matched for age, sex, chronic kidney disease stage and previous transient ischaemic attack (TIA), served as a comparison group. The main outcome was a composite of stroke, end-stage renal disease (ESRD) and death. Secondary outcomes included changes in myocardial left ventricular (LV) wall thickness and replacement fibrosis, change in glomerular filtration rate (GFR), new TIA and change in neuropathic pain.

**Results:**

During a median follow-up of 6.0 years (bottom and top quartiles: 5.1, 7.2), 15 events occurred in 13 patients (*n* = 7 deaths, *n* = 4 cases of ESRD and *n* = 4 strokes). Sudden death occurred (*n *= 6) only in patients with documented ventricular tachycardia and myocardial replacement fibrosis. The annual progression of myocardial LV fibrosis in the entire cohort was 0.6 ± 0.7%. As a result, posterior end-diastolic wall thinning was observed (baseline, 13.2 ± 2.0 mm; follow-up, 11.4 ± 2.1 mm; *P *< 0.01). GFR decreased by 2.3 ± 4.6 mL min^−1^ per year. Three patients experienced a TIA. The major clinical symptom was neuropathic pain (*n *= 37), and this symptom improved in 25 patients. The event rate was not different between the ERT group and the untreated (natural history) group of the Fabry Registry.

**Conclusion:**

Despite ERT, clinically meaningful events including sudden cardiac death continue to develop in patients with advanced Fabry disease.

## Introduction

Fabry disease is a rare X-linked lysosomal storage disorder caused by deficiency of the enzyme α-galactosidase A. The enzymatic deficit results in progressive intracellular accumulation of globotriaosylceramide in different tissues with profound clinical effects on the heart, kidneys and brain [1–6]. Storage of globotriaosylceramide starts before birth [7], and progressive organ failure leads to early death in hemizygous male patients, typically at the age of 40–50 years [8–11]. Enzyme-replacement therapy (ERT) with recombinant α-galactosidase A substitutes for the missing enzyme and is administered intravenously every 2 weeks. Two short-term clinical phase III trials have demonstrated that ERT is safe and well tolerated and is able to remove the microvascular deposits of globotriaosylceramide in biopsies of kidney, skin and heart of most patients with Fabry disease [12, 13]. A phase IV clinical trial over a median observation period of 18.5 months provided proof that ERT can slow the progression towards serious cardiac, renal and cerebrovascular complications [14]. Data from the Fabry Outcome Survey Registry on 5 years of treatment with ERT demonstrated a reduction in left ventricular hypertrophy and stabilization of kidney function in subgroups of patients [15]. However, the effects of long-term ERT on ‘hard’ clinical end-points including Fabry disease-related death remain unknown.

The aim of the current study was to examine the effects of ERT in patients with Fabry disease on progression towards the end-points long-term survival and cause-specific death, compared with the natural course of the disease.

## Methods

### Study population and protocol

Since the initiation of the Würzburg Fabry Disease Centre in 2001, 180 patients with genetically proven Fabry disease (73 male, 107 female) have been registered and are monitored regularly at the centre. In this cohort, ERT was initiated in 74 patients. Here, we report on the clinical course and outcome of all consecutive patients (*n *= 40) with genetically confirmed Fabry disease (for gene mutations see Supplementary Table S1) who have been treated for at least 5 years at a dose of 1 mg kg^−1^ body weight with recombinant α-galactosidase A (agalsidase beta; Fabrazyme, Genzyme, a Sanofi company, Cambridge, MA, USA) or died during the observation period. Patients were followed up to the time-point when they had to switch to agalsidase alpha because of agalsidase beta shortage due to viral contamination at manufacturing [16]. In general, most of these patients were relatively old and already advanced with respect to disease progression at baseline because ERT was not available before 2001. Thus, for these patients, this was the earliest opportunity to receive treatment with α-galactosidase A.

None of the patients had previously received infusions of recombinant α-galactosidase A. Before the first infusion (baseline) and at annual intervals, a complete medical history was taken, and cardiac, renal and neurological evaluations were performed. Here, we report data from the baseline and final visits. The cardiac and renal results after 1 and 3 years of observation have been reported previously [17–19].

Cardiac assessment was performed using echocardiography (morphology), strain rate (SR) imaging (regional myocardial function), 24-h Holter electrocardiogram (ventricular tachycardia) and magnet resonance imaging (MRI) with late enhancement (LE) imaging (myocardial fibrosis). Kidney function, that is, glomerular filtration rate (GFR), was measured by ^99^Tc-DTPA clearance, and proteinuria was assessed using 24-h urine collection.

The study conformed to the principles outlined in the Declaration of Helsinki. Written informed consent was obtained from all patients. The authors had full access to and take complete responsibility for the integrity of the data. Genzyme Corporation sponsors the Fabry Registry, maintains the database and has provided support for the analysis of the control group of patients from the Fabry Registry. All authors have read and approved the manuscript as written.

### Standard echocardiography

Left ventricular end-diastolic dimension as well as end-diastolic thickness of the posterior wall and the septum were measured using standard M-mode echocardiographic methods in parasternal long-axis images (GE Vingmed Vivid 7, Horten, Norway; 3.5 MHz). Left ventricular (LV) myocardial mass was calculated using the Devereux formula. Ejection fraction was calculated using the modified Simpson method. Blood pool pulsed Doppler of the mitral valve inflow was used to extract the ratio of early to late diastolic flow velocity and the deceleration time.

### Strain rate imaging

Real-time two-dimensional colour Doppler data were recorded from the interventricular septum and the LV lateral wall using standard apical four-chamber views to evaluate longitudinal function (GE Vingmed Vivid 7, Horten, Norway; 3.5 MHz). All data were analysed using dedicated software (Echopac®; GE Ultrasound, Horten, Norway). Longitudinal SR curves of the mid-apical septum and the basal–mid-lateral wall were extracted and peak systolic SR was derived. These longitudinal segments were chosen on the basis of experience gained in earlier studies of Fabry disease cardiomyopathy: fibrosis usually starts in the basal–mid-lateral wall and is not typically observed in the mid-apical septum [20–22]. All echocardiographic data were analysed blinded to the time-point of visit and to the degree of fibrosis.

### MRI

Routine MRI with gadolinium was carried out as part of the standard assessment for patients at the Würzburg Fabry disease centre. The LE technique (8 mm slice thickness, breath-hold, short heart axis) was applied to detect changes in tissue integrity in the LV myocardium [22]. Images were acquired using an inversion recovery sequence (field of view 240 × 320 mm^2^; matrix 165 × 256). Short axis views at the basal, mid and apical segments, covering the entire ventricle, were used to quantify the LE area (if present) by manual tracing. Applying this technique to a standard 17-segment model, every LV segment was evaluated for the occurrence of myocardial replacement fibrosis. A ratio of LE to complete LV cardiac mass was calculated [23]. All MRI data were analysed blinded to the time-point of visit.

### Definitions of outcome

The main outcome variable was defined as a composite of stroke, end-stage renal disease and initiation of dialysis and death.

Other cardiac outcomes were change in LV wall thickness (for morphology), change in peak systolic SR (for regional LV function), new ventricular tachycardia (for malignant arrhythmias) and change in LE-positive volume (for LV fibrosis). Kidney damage and kidney function were assessed by change in GFR and change in proteinuria. Neurological outcomes were defined as any new onset of transient ischaemic attack (TIA) or changes in neuropathic pain and hypohidrosis.

### Comparison group

After the approval of ERT as an efficacious and safe treatment option by the relevant authorities under the Orphan Drug Act, prospective randomized trials became largely impossible because of ethical considerations. Thus, we made use of the Fabry Registry (ClinicalTrials.gov Identifier: NCT00196742), an ongoing, international, multicentre, observational database that monitors natural history of the disease course and outcomes of patients with Fabry disease. The Fabry Registry began enrolling patients in April 2001 and included case records in the database for 4171 patients as of 6 January 2012 [24]. All patients with Fabry disease are eligible to enrol, regardless of age, gender, symptoms or treatment with ERT from any commercial source. We selected data from 40 adult subjects included in the Fabry Registry, matched to individuals in the treated group by year of birth, gender, previous TIA and chronic kidney disease (CKD) stage. These 40 patients were not treated with ERT because of problems with reimbursement in their countries of residence. Characteristics of this untreated comparison group are shown in Supplementary Table S2.

### Data analysis

Data are presented as mean ± standard deviation, median (bottom and top quartiles) or numbers (percentage), as appropriate. Baseline and follow-up values were compared using paired *t*-test or Fisher's exact test, as appropriate. For prognostic analyses, a Cox proportional hazard regression was used. The proportionality assumption was checked by visual inspection and no violation was found. Statistical interaction was examined by introducing the respective product term in the model. Predictors of the combined end-point were sought amongst the clinical characteristics shown in the tables, using a *P*-value of 0.1 in univariable analyses. For multivariable analysis, the backstep likelihood criterion was used (*P*_in_ = 0.05; *P*_out_ = 0.1). SPSS (version 19.0.1; SPSS Inc, Chicago, IL, USA) was used for statistical analysis.

## Results

### Patients

The baseline characteristics of all patients with Fabry disease, and subgroups with and without the main outcome, are presented in Table 1. The major clinical limitation at baseline was neuropathic pain, which was found in 37 patients. The 40 ERT-treated patients originated from 25 different families with Fabry disease. Only two families share the same stop codon mutation in exon 5 (for gene mutations see Supplementary Table S1). The mean α-galactosidase A activity assessed in white blood cells was 0.21 ± 0.12 nmol min^−1^ per mg in women and 0.03 ± 0.01 nmol min^−1^ per mg in men (the normal value is >0.4 nmol min^−1^ per mg). At baseline, 19 patients were treated with angiotensin-converting enzyme (ACE) inhibitors or angiotensin receptor blockers. During follow-up, all but six patients (with no documented proteinuria prior to or during the study) underwent titration of the ACE inhibitors or angiotensin receptor blockers.

**Table 1:** Baseline characteristics of all patients with Fabry disease

	All Pts (*n *= 40)	Pts with events (main outcome) (*n *= 13)	Pts without events (main outcome) (*n *= 27)
Age (years)	40 ± 9	45 ± 8	38 ± 9
Sex (male/female)	31/9	12/1	19/8
Weight (kg)	72 ± 12	72 ± 14	71 ± 10
Height (cm)	175 ± 9	178 ± 7	174 ± 9
Heart rate (min^−1^)	64 ± 10	64 ± 11	65 ± 11
Systolic BP (mmHg)	127 ± 15	126 ± 17	127 ± 15
Diastolic BP (mmHg)	79 ± 11	80 ± 11	78 ± 11
Atrial fibrillation (*n*, %)	3 (8)	2 (15)	1 (4)
Angina pectoris (*n*, %)	13 (33)	7 (54)	6 (22)
Dyspnoea (*n*, %)	19 (48)	7 (54)	12 (44)
Glomerular filtration rate (mL min^−1^)	85 ± 28	61 ± 25	94 ± 22
Proteinuria (g)	0.63 ± 0.96	2.4 ± 2.2	0.5 ± 0.6
Dialysis (*n*, %)	7 (18)	4 (31)	3 (11)
Kidney transplantation (*n*, %)	4 (10)	2 (15)	2 (7)
Stroke (*n*, %)	3 (8)	3 (23)	0 (0)
TIA (*n*, %)	3 (8)	0 (0)	3 (11)
Neuropathic pain (*n*, %)	37 (93)	11 (85)	26 (96)
Hypohidrosis (*n*, %)	33 (83)	11 (85)	22 (82)

Pts, patients; BP, blood pressure; TIA, transient ischaemic attack.

Data are given as mean ± SD or numbers (%).

### Main outcome

**Fig 1:**
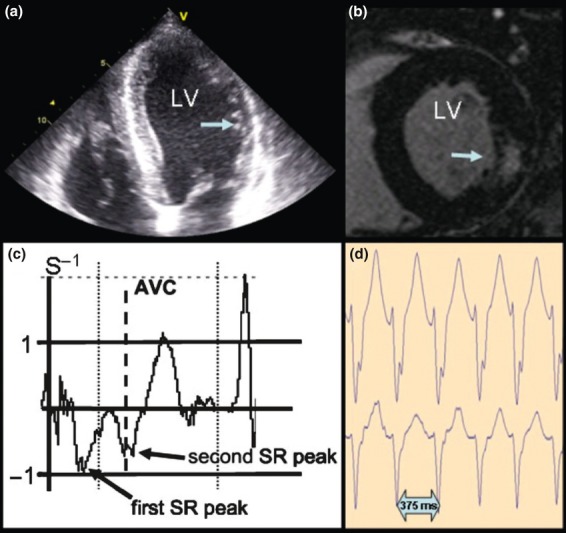
Example of data from a typical patient with Fabry disease who died during follow-up. (a) Echocardiographic four-chamber view with hypertrophy of the left ventricular septum and thinning of the lateral wall (arrow). (b) Magnetic resonance image showing short axis view with a late enhancement-positive segment in the posterolateral wall (arrow) of the left ventricle. (c) Strain-rate curve (for the evaluation of regional myocardial function) extracted from the basal left ventricular lateral wall over one heart cycle showing reduced regional myocardial function (reduced first systolic strain-rate peak) and a second strain-rate peak shortly after aortic valve closure as a typical sign of myocardial fibrosis. (d) Section of a 24-h Holter electrocardiogram showing a typical ventricular tachycardia with a heart rate of 160 beats per min. AVC, aortic valve closure; LV, left ventricle; SR, strain rate.

The median follow-up time for survivors treated with ERT was 6.0 years (bottom and top quartiles: 5.1, 7.2). There were 15 events (death, stroke and end-stage renal disease) in 13 patients (Table 1). Seven patients (all male) died during follow-up due to infection (*n *= 1) or sudden cardiac death (*n* = 6) at a mean age of 53 ± 6.8 years (range 42–61 years). These seven patients had received ERT on average for 60 ± 32 months (range 10–104 months). One patient had received an implantable cardioverter defibrillator (ICD) 2 years prior to death, with several documented episodes of ventricular tachycardia adequately abolished by the device. All patients who died because of cardiac arrest had ventricular tachycardia according to Holter ECG. Four had a sustained and two a nonsustained episode. In one of these patients, ventricular tachycardia was recorded by online monitoring during multiple in-hospital resuscitations. According to echocardiographic appearance, all six patients had hypertrophic cardiomyopathy and demonstrated at least two LE-positive segments *loco typico*, indicating advanced myocardial fibrosis (Fig. 1). One patient experienced a fatal event due to sepsis, which is not typical of Fabry disease. Prior to death, one patient had a stroke and another had a stroke prior to end-stage renal disease (Table 2).

**Table 2:** Baseline characteristics of all patients with Fabry disease with a main outcome event

Patient (sex)	Type of event	Age at ERT start (years)	GFR at baseline (mL min^−1^)	ERT Time to event (months)
1 (male)	Sepsis and death	41	81	12
2 (male)	Cardiac death	45	81	75
3 (male)	Cardiac death	46	ESRD^a^	10
4 (male)	Cardiac death	51	81	77
5 (male)	Cardiac death	51	ESRD^a^	72
6 (male)	Cardiac death	53	48	104
7 (male)	Cardiac death and stroke	39	ESRD^a^	68 15
8 (male)	Stroke	54	43	6
9 (female)	Stroke	56	95	42
10 (male)	Stroke and ESRD	41	47	70 72
11 (male)	ESRD	29	81	46
12 (male)	ESRD	37	22	72
13 (male)	ESRD	38	33	35

ERT, enzyme-replacement therapy; GFR, glomerular filtration rate; ESRD, end-stage renal disease.

a Patients with ESRD at baseline were treated with maintenance haemodialysis.

Four other patients (all male) progressed to end-stage renal disease and initiation of dialysis at the ages of 33, 41, 43 and 47 years. Three of the four already had moderate to severe CKD (GFR of 22, 33 and 47 mL min^−1^) and proteinuria (2.3, 2.3 and 6.6 g per day) at first presentation; haemodialysis was started after 3, 6 and 6 years on ERT. The last of the four patients progressed to dialysis within 4 years declining from a baseline GFR of 81 mL min^−1^ associated with proteinuria (3.1 g per day) (Table 2).

Four patients (three male and one female) developed a stroke during follow-up at age 41, 46, 54 and 60 years, respectively (Table 2). Only one of these patients experienced a TIA before the onset of stroke; all had myocardial fibrosis and advanced cardiomyopathy. Subsequent thrombo-embolism due to atrial fibrillation was suspected in two of the four patients.

In univariate Cox regression analysis, the following variables were associated with the occurrence of the combined end-point [hazard ratio (HR); 95% confidence interval (CI)]: LE (5.17; 1.13–23.62), cardiac mass (per 10 g: 1.12; 1.03–1.23), GFR (per mL min^−1^: 0.98; 0.97–0.99), previous cerebrovascular accident (4.36; 1.14–16.64), New York Heart Association (NYHA) functional class (per class: 2.22; 0.93–5.26) and proteinuria (per 1 g: 1.31; 0.97–1.75). In multivariable analysis, only NYHA functional class (per class: 2.74; 1.13–6.66; *P *= 0.025) and proteinuria (per 1 g: 1.47; 1.05–2.05; *P *= 0.026) remained independently predictive.

### Secondary outcomes

The organ-specific outcomes (cardiac, renal and cerebral) are summarized in Table 3. The last available information is reported for patients who died.

**Table 3:** Organ-specific outcomes (*n *= 40)

	Baseline	Follow-up	*P*-value*
Heart			
Septum (mm)	13.5 ± 2.0	11.9 ± 1.8	<0.0001
PWT (mm)	13.2 ± 2.0	11.4 ± 2.1	<0.0001
LV mass (g)	270 ± 87	224 ± 71	<0.0001
LVEDD (mm)	49 ± 5	49 ± 6	0.883
EF (%)	64 ± 6	63 ± 7	0.692
E/A	1.3 ± 0.4	1.2 ± 0.4	0.061
DT (ms)	225 ± 60	217 ± 63	0.383
SR septum (s^−1^)	1.3 ± 0.2	1.4 ± 0.3	0.365
SR lateral (s^−1^)	0.9 ± 0.3	0.7 ± 0.3	<0.0001
Pts with fibrosis (*n*)	29	31	0.797
Pts with new fibrosis (*n*)	–	2	–
Fibrosis in relation to LV (%)	1.7 ± 2.7	3.7 ± 4.0	<0.0001
Pts with new VT (*n*)	–	12	–
Kidney			
GFR (mL min^−1^)	85 ± 28	73 ± 39	0.003
Proteinuria (mg per day)	633 ± 961	209 ± 215	0.018
Brain			
New TIA (*n*)	–	3	–
Any neuropathic pain (*n*)	37	21	0.0001
Pts with improved pain (*n*)	–	25	–
Analgesic medication (*n*)	16	16	0.999
Hypohidrosis (*n*)	33	24	0.046
Pts with improved hypohidrosis (*n*)	–	14	–

PWT, posterior wall thickness; LV, left ventricle; LVEDD, left ventricular end-diastolic diameter; EF, ejection fraction; E/A, ratio of early to late diastolic flow velocity; DT, deceleration time; SR, strain rate; Pts, patients; VT, ventricular tachycardia; GFR, glomerular filtration rate; TIA, transient ischaemic attack.

The last value available from the latest follow-up was carried forward for patients who died.

* Paired *t*-test or Fisher's exact test, as appropriate.

Both septal and posterior end-diastolic wall thickness decreased significantly, but longitudinal systolic SR increased in the septum (not significantly) and decreased significantly in the lateral wall. Two patients developed a new LE-positive segment *loco typico*. The annual progression of LV fibrosis was 0.6 ± 0.7% (men: 0.7 ± 0.7%; women: 0.2 ± 0.3%). During follow-up, four patients received an ICD because of sustained (*n *= 3) or nonsustained (*n *= 1) ventricular tachycardia. Subsequent episodes of adequately terminated ventricular tachycardia were recorded by the ICDs in three of these four patients. Two other patients who exhibited nonsustained ventricular tachycardia were scheduled to receive implantation of an ICD after the observation period had ended (i.e. at the time the dosage was decreased or they switched to another source of ERT). Almost all patients with documented ventricular tachycardia had hypertrophic cardiomyopathy with myocardial fibrosis, but ventricular tachycardia was never detected in patients without LE.

During follow-up, GFR decreased by 2.3 ± 4.6 mL min^−1^ per year (men: 2.4 ± 5.0 mL min^−1^ per year; women: 1.9 ± 3.3 mL min^−1^ per year). In patients without proteinuria (<150 mg per day) at baseline (*n *= 14), GFR remained stable over time (baseline 88 mL min^−1^; follow-up 88 mL min^−1^). Four out of the five patients who had proteinuria >2 g per day at baseline progressed to end-stage renal disease. Kidney function in the remaining patient also worsened and GFR decreased from 48 to 27 mL min^−1^.

Only three patients developed a TIA during follow-up. Neuropathic pain was frequently observed at baseline (*n *= 37), but improved markedly in 25 patients. Likewise, hypohidrosis improved in 14 patients, but to a lesser degree.

### Natural course of Fabry disease

**Fig 2:**
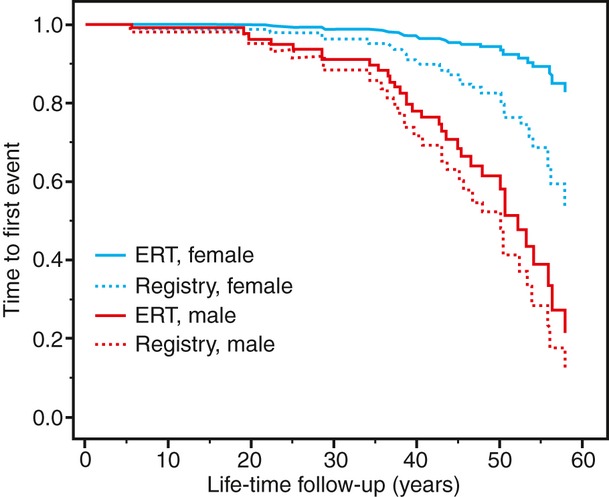
Incidence of stroke, haemodialysis or death in 40 subjects treated with enzyme-replacement therapy (ERT) for a period of at least 5 years (ERT group) versus 40 subjects from the Fabry Registry matched for year of birth, sex, chronic kidney disease stage and previous transient ischaemic attack. Unadjusted graphs from Cox regression plots are shown for both sexes (*n = 9 women per group)*.

Nine of the 40 matched ERT-naïve patients from the Fabry Registry were female (23%). Further characteristics of this comparison group are shown in Supplementary Table S2. During the median life-time follow-up of 45 years (bottom and top quartiles: 38, 52 years), 10 patients developed a stroke (25%), 11 patients (28%) progressed to end-stage renal disease with initiation of dialysis and two patients died (5%). Because two patients experienced several events, only 21 events that occurred in more than half of the cohort were included in the main outcome analysis. Over a median life-time follow-up 44 years (quartiles: 39, 51 years), there was no significant difference in the main outcome between the control group (natural course of the disease) and the ERT group in Cox regression adjusted for sex (HR 1.48; 95% CI 0.72–3.06; *P *= 0.284). Figure 2 shows time to first event (stroke, renal death or all-cause mortality for the ERT and control Registry groups) stratified by sex; life-time follow-up was derived from Cox regression analysis. No interaction between group and sex was found (*P *> 0.3).

## Discussion

Recombinant human alpha-galactosidase A therapy for Fabry disease received orphan drug designation approval in 2001 after clinical studies demonstrated tissue clearance of the substrate and symptom relief in patients [12, 13]. Subsequent clinical studies focused on organ and heart function and identified groups of patients with nonuniform responses to treatment [14, 15, 17, 19, 24, 25]. The success of treatment was dependent on the severity of organ involvement and was essentially a function of age [14, 15, 19]. Here, we report disease progression beyond cardiac and renal damage occurring in 35% of the present cohort after 6.0 years of uninterrupted treatment with the licensed dose of algasidase beta. The main finding in this patient cohort was sudden cardiac death, explained by progressive replacement fibrosis in the heart and subsequent ventricular arrhythmia. We postulate that a similar mechanism accounts for the documented progression to renal death and stroke. Thus, ERT could not fully prevent development of fibrosis and organ failure in more than a third of this cohort with Fabry disease during a period of nearly 6 years.

The safety and efficacy of ERT were shown in two initial studies [12, 13]. Eng *et al*. [12] demonstrated in a randomized, double-blind, placebo-controlled study that ERT resulted in histological clearance of the deposits of globotriaosylceramide from the heart, kidney and skin. Subsequently, clinical short-term follow-up studies in relatively small cohorts or registries of patients with Fabry disease focused on the impact of ERT on organ function. These studies also showed an improvement in Fabry disease-related symptoms, and the results suggested a stabilization of kidney function and a reduction in LV hypertrophy [17–19, 24, 25]. Similarly, the current long-term follow-up study confirms that typical Fabry disease-related symptoms such as neuropathic pain and hypohidrosis improve over time in most patients. Focusing on organ-related outcomes, it was shown in a randomized controlled trial with a median observation time of 18.5 months that ERT slowed the progression towards cardiac, renal and cerebrovascular complications [14]. It is interesting that in this trial in patients with advanced renal involvement, ERT had no impact on progression of the disease, whereas substantial improvement could be observed in those with mild renal involvement [14]. Furthermore, Germain *et al*. provided proof that, amongst patients with Fabry disease, a subpopulation with impaired renal function (reduced GFR, proteinuria and glomerulosclerosis) at baseline has a less favourable outcome and may develop renal progression despite treatment with ERT [26, 27]. By contrast, using natural history data from the Fabry Outcome Survey database (sponsored by Shire Human Genetic Therapies, Lexington, MA, USA), it was shown that pain and quality of life improved, LV mass decreased and the rate of loss of kidney function declined after 5 years of ERT [15].

Finally, the results of the current systematic long-term follow-up study, focusing primarily on cardiac death, renal death and stroke demonstrate that the three main target organs of Fabry disease cannot be prevented from failing, despite ERT. Thus, in the advanced stages of Fabry disease, specific treatment can decrease symptoms, but not prevent progression towards organ damage and subsequently death. Slowing the progression of the disease by ERT may still be an important option, given the heterogeneity of disease development between men and women and even amongst individuals within the same families. It is possible that because the present cohort is older than previously studied patient groups and thus at a later stage of disease, ERT may be less effective. The average age of patients who reached the stage of renal replacement therapy was 33–47 years. Patients who died were 42–61 years old; this is similar to the age range of those who experienced a stroke: progression of this orphan disease appears to be a function of age without any subtle slowing of development with treatment. Early treatment, that is, as early as possible during adulthood, may be recommended, but treatment success may only be recognized, if at all, after decades of ERT. Potentially, we see progression of the disease as a function of age without the chance to detect subtle changes of retardation in this orphan disease. Advocating earlier treatment, that is, as early as possible during adulthood, may be the recommendation of choice, but similarly we could evaluate treatment success, if at all, only after decades of treatment.

It has been demonstrated previously that ERT improves regional myocardial function and reduces LV hypertrophy during the first 3 years of treatment [15, 18, 19, 24]. Patients in the present study also showed a significant reduction in septal and posterior wall thickness. We propose that a decrease in septal wall thickness is required for treatment to be considered successful. Takenaka and coworkers suggested that a reduction in posterior wall thickness probably reflects myocardial fibre dropout and wall thinning due to progressive fibrosis with subsequent reduction in regional LV myocardial function [28]. Despite ERT, the annual progression of LV fibrosis was 0.7 ± 0.7% in men and 0.2 ± 0.3% in women. Subsequent development of malignant ventricular tachycardia appears to be the consequence of ongoing myocardial fibrosis, which is consistent with the findings of the autopsy study by Takenaka *et al*. [28]. Of interest, ventricular tachycardia was not detected in any patients without LV fibrosis.

Results from previous trials have demonstrated that ERT has no impact on proteinuria and improvement of kidney function [14, 15, 17, 26, 27, 29–31]. In the present study, the annual decrease in GFR was 2.3 ± 4.6 mL min^−1^ resulting in substantial loss of kidney function over time. Intense antiproteinuric therapy through blockade of the renin–angiotensin system (RAS) is recommended to reduce proteinuria [31]. Despite ERT, we could not prevent end-stage renal failure in most patients with baseline proteinuria above 2 g per day. Nevertheless, RAS blockade is advisable in patients with Fabry disease with overt proteinuria.

Cryptogenic stroke is an established complication of Fabry disease [2, 32–34]. The current findings suggest that ERT cannot prevent TIA or strokes. Only one treated patient developed a TIA before a stroke. Our data suggest that it is important to focus on severe neurological complications as a consequence of the severity of cardiomyopathy and cardiac thrombo-embolism. Diagnostic procedures should rule out atrial fibrillation on a routine basis in order to prevent strokes of cardiac origin because of the availability of preventive measures such as anticoagulation.

The current findings indicate that patients with advanced Fabry disease should undergo a comprehensive organ staging at routine intervals, as recommended by current guidelines [16, 35]. Experience from patients with other chronic progressive diseases may be applied to those with Fabry disease. The amount of proteinuria and the level of myocardial replacement fibrosis are helpful prognostic indicators to motivate the initiation of adjunctive therapies to prevent major complications of Fabry disease.

Although the observation period was long, the size of the cohort was relatively small. Registries would provide the opportunity to collect solid outcome data from larger groups, but the comprehensive collection of data from LE imaging, SR imaging, assessment of ventricular tachycardia and tracer studies of renal function is a major challenge. Nevertheless, the follow-up time of 6 years may compensate partly for the sample size as at least one major event was recorded in 35% of patients.

In the current study, it was not possible to include a randomized control group. Because of ethical considerations as stated above, patients at a similar stage of disease could not be deprived of a therapy considered to be effective. To compensate, we used carefully matched registry data from subjects following the natural course of the disease; however, due to the observational nature of the data, careful interpretation is recommended.

It was initially intended that our cohort study comprising 180 individual patients would continue for longer than 6 years. The advent of enzyme shortage from September 2009 forced us to terminate the study as most patients were initially treated with low-dose agalsidase beta and were then switched to licensed doses of agalsidase alfa. The present data may have an important impact when the supply of agalsidase beta is restored and triage strategies can provide enzyme to those who need it most.

In conclusion, ERT in patients with advanced Fabry disease did not prevent progression towards fatal organ failure and death. These results support the rationale for early treatment of patients with Fabry disease.

## Conflict of interest statement

F. Weidemann, M. Niemann and F. Breunig have received speaker honoraria from Genzyme and Shire Corporation. F. Weidemann and C. Wanner are members of the Fabry Registry European Board of Advisors and have received travel assistance and speaker honoraria. Research grants were given by Genzyme and Shire Corporation.
