# Supplementary material for: Long-term outcome of enzyme-replacement therapy in advanced Fabry disease: evidence for disease progression towards serious complications
Source: J Intern Med. 2013 May 6;274(4):331–41. doi: 10.1111/joim.12077 (PMC4282332; doi:10.1111/joim.12077)
Supplement: Supplementary file 1 [file joim0274-0331-SD1.docx]

**Supplemental Table 1: Table of alpha-galactosidase A mutations**

C63Y

G35R (2x)

p.162del (2x)

R112C

IVS2+1TG>a

D136E (2x)

IVS3+1 G>A (3x)

A135V

IVS3(-1) (2x)

IVS0 (-10)

L129P

D165V

fs191x

fs248x (2x)

R227X (2x)

W236C

Asn215Ser

IVS6+10

W349X

W399X (2x)

354-359del

Fs417X (2x)

E341K (2x)

R342L (4x)

p.1208del (2x)

**Supplemental Table 2: Demographic, renal and cardiac characteristics of patients of the Fabry Registry**

|  | Patients representing the natural course of the disease, matched for the present study | | Other patients of the Fabry Registry | |
| --- | --- | --- | --- | --- |
|  | Males  N=31 | Females  N=9 | Males  N=1,946 | Females  N=2,072 |
| Current age (yrs) | 46.5 (26.7, 63.9) | 52.5 (42.0, 74.4) | 40.8 (0.5, 85.7) | 44.4 (2.1, 90.9) |
| Age at diagnosis (yrs) | 38.0 (10.7, 62.1) | 39.3 (6.1, 66.9) | 25.8 (0.0, 81.0)* | 32.9 (0.0, 82.4)* |
| Age at last follow-up (yrs) | 45.3 (24.0, 63.8) | 51.3 (41.1, 74.1) | 39.3 (0.1, 84.8) | 42.5 (0.6, 85.7) |
| Pre-treatment CKD stage 1-5, (%) | 36 / 17 / 13 / 7 / 27 | 56 / 33 / 11 / 0 / 0 | 51 / 22 / 15 / 5 / 7 | 60 / 30 / 8 / 1 / 1 |
| Pre-treatment UPCR category, % | 20 / 20 / 50 / 10 | 50 / 50 / 0 / 0 | 45 / 22 / 24 / 9 | 59 / 19 / 15 / 7 |
| Pre-treatment LPWT >12 mm, % | 60 | 100 | 47 | 26 |

* Patients in whom Fabry disease was diagnosed pre-natally were coded as age at diagnosis = 0 yrs.

Age at last follow-up defined as the age at the last reported assessment (or age at death if deceased).

CKD: chronic kidney disease stage according to estimated glomerular filtration rate (eGFR in ml/min/1.73 m^2^) calculated by the CKD-EPI formula; stage 1: eGFR ≥90, stage 2: ≥60-<90, stage 3: ≥30-<60, stage 4: ≥15-<30, stage 5: <15; information available for all “matched patients” and 2,740 “other patients” (1,219 males and 1,521 females).

UPCR: urine protein to creatinine ratio was calculated using both 24-hour and spot values when creatinine was available on the same day as the protein value; values were categorized as <0.3 g/g; ≥0.3-<1 g/g; ≥1-<3 g/g; ≥3 g/g; information available for 14 “matched patients” (10 males and 4 females) and 1,620 “other patients” (655 males and 965 females).

LPWT. left ventricular posterior wall thickness; information available for 26 “matched patients” (20 males and 6 females) and 2,161 “other patients” (868 males and 1,293 females).

Last record value was used in untreated patients and last record before initiation of first treatment was used for treated patients.
